# Bottom-Up Interventions Effective in Promoting Work Engagement: A Systematic Review and Meta-Analysis

**DOI:** 10.3389/fpsyg.2021.730421

**Published:** 2021-09-08

**Authors:** Janina M. Björk, Pernilla Bolander, Anna K. Forsman

**Affiliations:** ^1^Department of Developmental Psychology, Faculty of Education and Welfare Studies, Åbo Akademi University, Vaasa, Finland; ^2^Department of Management and Organization, Stockholm School of Economics, Stockholm, Sweden; ^3^Department of Health Sciences, Faculty of Education and Welfare Studies, Åbo Akademi University, Vaasa, Finland

**Keywords:** work engagement, workplace resources, bottom-up approaches, workplace interventions, systematic review, meta-analysis

## Abstract

**Background:** Promoting work engagement is of interest to organizations across sectors due to the associated positive outcomes. This interest warrants research on the evidence of work engagement interventions. Intervention research increasingly advocates a bottom-up approach, highlighting the role of employees themselves. These workplace interventions often encourage employees to identify, develop, and make use of workplace resources. The aim of this systematic review and meta-analysis is to investigate the effectiveness and potential underlying mechanisms of these bottom-up, resource-developing interventions.

**Method:** Systematic searches were conducted in the online databases Web of Science, Academic Search Complete, Business Source Ultimate, PsycInfo, PsycArticles, SCOPUS, and Google Scholar. Publication year range was 2000–2020. Eligibility criteria were defined using PICOS. To be eligible for the systematic review, the intervention study identified had to aim at promoting working individuals’ work engagement by developing workplace resources from bottom-up. Work engagement had to be measured using the Utrecht Work Engagement Scale. The systematic review included one-, two-, or multiple-armed – randomized or non-randomized – intervention studies with various study designs. Further, a meta-analysis was conducted on a sub-set of the studies included in the systematic review. To be eligible for the meta-analysis, the studies had to be two- or multiple-armed and provide the information necessary to compute effect sizes.

**Results:** Thirty-one studies were included in the systematic review. The majority reported that overall work engagement increased as an effect of the intervention. The evidence regarding the sub-components of work engagement was scattered. Potential underlying mechanisms explored were intervention foci, approach, and format. Dimensions of satisfaction and performance were identified as secondary outcomes. Participant experiences were generally described as positive in most of the studies applying mixed methods. The meta-analysis showed a small but promising intervention effect on work engagement (24 studies, SMD: −0.22, 95% CI: −0.34 to −0.11, with *I*^2^=53%, indicating moderate inconsistency in the evidence).

**Conclusion:** The synthesized evidence suggests that bottom-up, resource-developing interventions are effective in the promotion of work engagement. The meta-analysis suggests that focusing on strengths use or mobilizing ego resources and adopting a universal approach increase intervention effectiveness.

## Introduction

Ever since Kahn’s (1990) seminal paper on “personal engagement” at work was published, the promotion of engagement has attracted the attention of scholars and practitioners alike. Given its well-documented association with outcomes of great value at the workplace, such as employee wellbeing and work performance (e.g., Bakker and Bal, 2010; Christian et al., 2011; Bailey et al., 2017), the interdisciplinary interest in engagement shows no signs of decline.

Numerous conceptualizations, definitions, and measures of engagement have emerged (for reviews, see Bailey et al., 2017; Shuck et al., 2017; Kelders et al., 2020). However, in the present study, we conceptualize engagement as work engagement: a positive, psychological state consisting of the three subcategories vigor, dedication, and absorption (Schaufeli et al., 2002). This definition of work engagement, provided by the Utrecht Group, is widely accepted. The measurement scale developed by the same research team [Utrecht Work Engagement Scale (UWES; Schaufeli et al., 2006)] is also extensively adopted (Bailey et al., 2017; Shuck et al., 2017; Kelders et al., 2020). Although some researchers (e.g., Wefald et al., 2012) have criticized this scale, its validity and reliability are supported by a strong evidence base (Schaufeli, 2014). In these two respects, the work engagement research domain is considered mature and intervention research is increasingly warranted (e.g., Leiter and Maslach, 2010).

A wide range of work engagement interventions is emerging, spanning from interventions focused on developing workplace resources (e.g., Bakker and van Wingerden, 2020), to interventions aimed at developing leaders (e.g., Biggs et al., 2014) and promoting healthy lifestyles (e.g., Strijk et al., 2013). In a rough sense, these interventions take either a top-down or a bottom-up approach. Whereas top-down interventions are initiated and driven by senior management, often with the intention to create organization-wide effects, bottom-up interventions are initiated and driven by employees and aim to make changes that have effects on the employees themselves and their immediate work environment (Hornung et al., 2010). Importantly, different factors are purported to impact the effectiveness of work engagement interventions depending on what changes are being made and by whom. Therefore, the conclusions drawn from one type of intervention may not be directly transferable to and comparable with other types. In the present systematic review and meta-analysis of work engagement interventions, we thus narrow our focus with respect to what changes are being made and by whom, which enables us to delve into the effectiveness and mechanisms underlying interventions of the same type.

First, we focus on work engagement interventions aimed at developing workplace resources. Research on workplace resources has expanded rapidly during the past two decades due to the growing influence of theoretical frameworks, such as the conservation of resources (COR) theory (Hobfoll, 1989; Halbesleben et al., 2014), the job demands-resources model (JD-R; Demerouti et al., 2001), and the broaden-and-build theory (Fredrickson, 2001). In the present study, resources are broadly defined as “anything perceived by the individual to help attain his or her goals” (Halbesleben et al., 2014, p. 5). Following Nielsen et al. (2017), we focus specifically on workplace resources in this study, that is, resources that help individuals to attain their work-related goals and promote their work engagement. Workplace resources can be inherent in the working individuals themselves (e.g., self-efficacy, hope, optimism, and resilience), reside in their social context (e.g., supervisor and social support, team climate, and group-person fit), or be afforded by the way work is organized, designed, or managed (e.g., autonomy, skills variety, and job control; Nielsen et al., 2017). Hence, workplace resources are to a large extent psychosocial by nature and emerge from the interaction between the working individual and the workplace (Su et al., 2021).

Second, we limit our focus to work engagement interventions with bottom-up approaches. A growing number of scholars argue that organizations increasingly have to rely on employees’ proactive behavior and engagement as working life is becoming more dynamic and organizations have less time to create resourceful work environments for their employees (e.g., Grant and Ashford, 2008; Bakker et al., 2012; Bakker, 2017). Consequently, it has been suggested that organizations can facilitate and support employees in developing workplace resources for the promotion of work engagement by offering interventions in which employees learn, practice, and implement individual bottom-up strategies. Bakker (2017) suggests four individual bottom-up strategies that can be taught: self-management, job crafting, strengths use, and mobilizing ego resources.

The current evidence base on the effectiveness of interventions aimed at promoting work engagement, in which employees themselves are encouraged to develop workplace resources, is limited. Some prior studies have taken a broader approach than the study at hand. A few narrative syntheses of the engagement literature focus on conceptual issues and on explaining the meaning, antecedents, and outcomes of various forms of employee engagement, not specifically targeting the work engagement domain (e.g., Bailey et al., 2017; Shuck et al., 2017; Kelders et al., 2020). A previous narrative synthesis (Knight et al., 2019) and a systematic review with meta-analysis (Knight et al., 2017) both assess the overall effectiveness of a wide range of work engagement interventions (e.g., top-down and bodily health-focused interventions). In another study, Nielsen et al. (2017) systematically review and meta-analyze studies with various research designs (such as cross-sectional and longitudinal) focused on workplace resources to promote general employee wellbeing (e.g., work engagement, happiness, and job satisfaction) and performance. Other prior studies have taken a narrower approach than the study at hand. Specifically, prior meta-analytic studies on bottom-up interventions to promote work engagement narrow their focus to job crafting, thereby excluding other bottom-up strategies, such as mindfulness. Further, these meta-analyses evaluate additional outcomes to work engagement, such as job crafting behavior and work performance (Oprea et al., 2019), or include studies other than interventions, such as longitudinal and daily diary studies (Rudolph et al., 2017; Frederick ad VanderWeele, 2020). In conclusion, previous review exercises on work engagement research have either been broader or narrower in their scope than the current study. To the authors’ knowledge, the effectiveness of and the underlying mechanisms to effective interventions aimed at promoting work engagement by developing workplace resources from bottom-up have not yet been systematically reviewed and meta-analyzed.

### Objectives and Research Questions

The aim of the present study was to conduct a systematic review and meta-analysis to synthesize the evidence base of interventions focused on promoting work engagement by developing workplace resources from bottom-up. It is our hope that the findings will guide not only future work engagement research and practice, but also that of the broader organizational psychology field. Specifically, we addressed the following research questions:

What is the evidence base for the effectiveness of bottom-up, resource-developing interventions targeting employees in the promotion of work engagement?

- Based on the systematic review and meta-analysis, what is the evidenced effectiveness of the identified interventions for work engagement (primary outcome)? What does the evidence say about other employee outcomes measured (secondary outcomes)?
- What study design is applied in the evidence-based work engagement interventions identified?
- What are the potential mechanisms underlying the evidence-based work engagement interventions identified?

## Materials and Methods

### Study Protocol

We conducted the current study in accordance with the guidelines presented in the Preferred Reporting Items for Systematic Reviews and Meta-analyses (PRISMA) statement (Moher et al., 2009) to the extent that they apply to non-medical research. These guidelines include following a checklist for reporting (see Supplementary Data Sheet S1). Our study approach (e.g., search strategies and data extraction) was also consistent with that of ample review exercises on work engagement published in the past (e.g., Knight et al., 2019).

### Search Strategy

Our comprehensive search strategy included searches in seven international, scientific online databases, chosen with regard to the interdisciplinary nature of the research topic. Four of these were specialized EBSCO databases: Academic Search Complete, Business Source Ultimate, PsycInfo, and PsycArticles. The three additional online databases that we conducted searches in were Web of Science, SCOPUS, and Google Scholar. We included research published between January 2000 and December 2020. The main searches in databases were conducted between September 25 and October 14, 2020, and the same searches were repeated on February 22–23, 2021 in order to include records from the end of year 2020. The selected databases along with database-specific search strategies are described in detail in the supplemental material (see Supplementary Data Sheet S2).

In accordance with the standard PICOS approach (Participants, Interventions, Comparisons, Outcomes, and Study design; Moher et al., 2009), we defined the following eligibility criteria for the systematic review:

- intervention population target group was working individuals in any industry or organizational context worldwide;
- interventions were aimed at developing workplace resources from bottom-up (Hornung et al., 2010; Bakker, 2017);
- comparators, if any, were groups receiving no-intervention (i.e., waiting list and inactive) and/or other intervention;
- the primary outcome was overall work engagement or one of its sub-components (i.e., vigor, dedication, or absorption) and measured using the short or long version of the UWES-scale (Schaufeli and Bakker, 2003; Schaufeli et al., 2006);
- the study design was quantitative (one-, two-, or multiple-armed intervention studies with randomized or non-randomized allocation of participants), qualitative (e.g., interviews), or mixed (i.e., quantitative and qualitative study design combined).

Additionally, we adopted eligibility criteria relevant to our systematic review but not specified in PICOS. Specifically, these criteria were that the included studies should be published in peer-reviewed established journals (i.e., journals with an impact factor, not conference papers, dissertations, or books); written in English; focused on the promotion of work engagement (i.e., not focused on how to prevent decreased work engagement); and the presented study findings should be based on completed intervention studies (i.e., not study protocols). We included intervention studies in which individual bottom-up approaches and individual-level outcomes were in focus (i.e., participatory action interventions and/or aggregated outcome measures were not considered), although the interventions included could be delivered in various ways (e.g., target groups of employees, individual employees, and online or face-to-face). Due to the psychological nature of the review primary outcome, we excluded studies that emphasized physiological resources related to lifestyle and bodily health (e.g., low blood pressure, yoga, and diet), rather than psychosocial resources related to the interaction between the individual and the workplace (which can be inherent in the individual, reside in the social context, or in the way work is organized). Since the target population was working individuals, we excluded studies focusing on the work engagement of other groups of individuals (e.g., students). No limitations were applied regarding the duration of the intervention program.

The meta-analysis was conducted on a sub-set of the studies included in the systematic review. To be eligible for the meta-analysis, the studies had to include a control group (i.e., waiting list, inactive, or other intervention) and provide eligible information to compute pooled effect sizes (alternatively information retrievable from other sources than the actual report).

### Study Selection and Data Extraction

**Figure 1:**
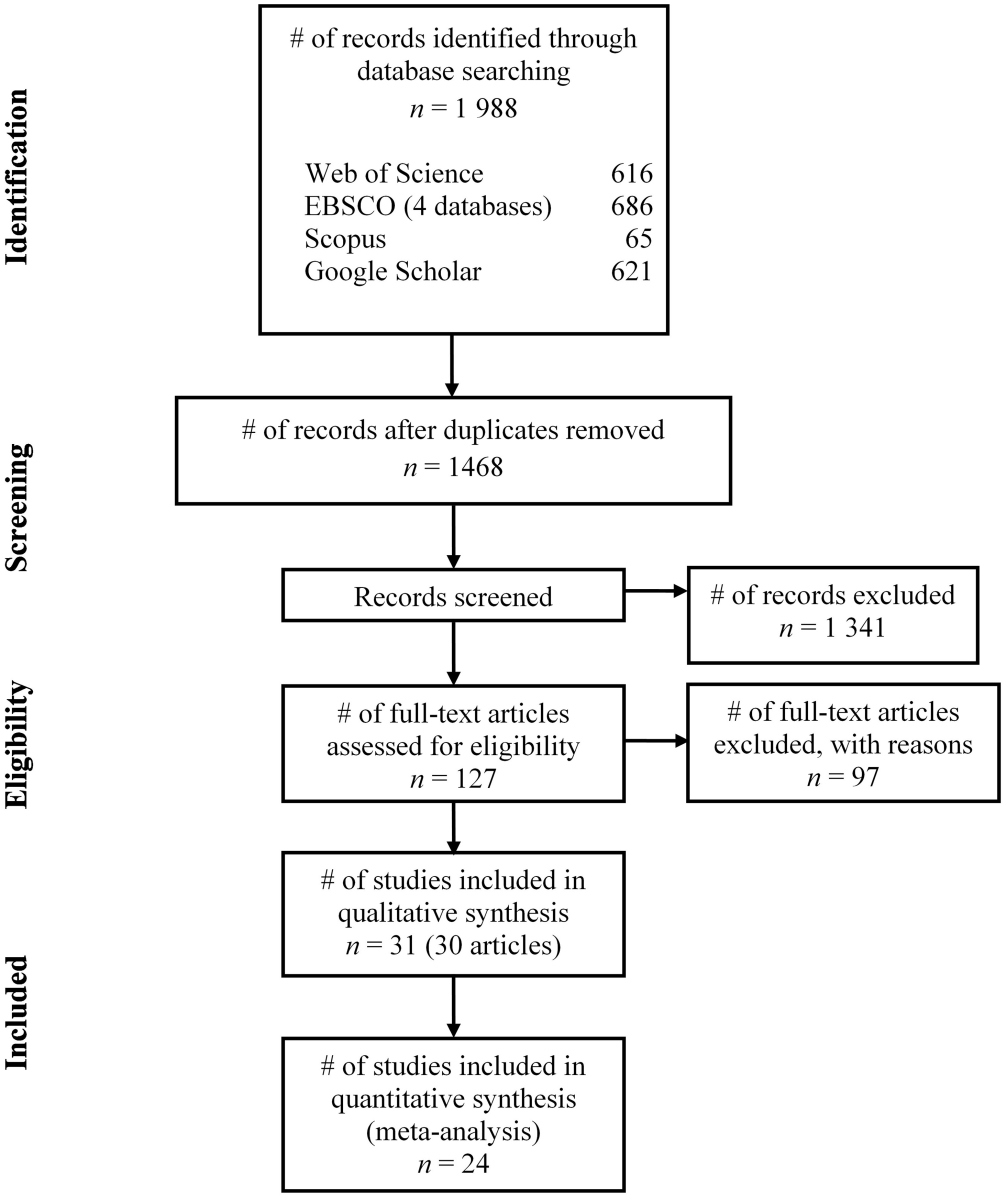
PRISMA flow diagram illustrating the conducted screening and selection process. *Source:* Moher et al. (2009).

The first author managed the abstract screening process independently. The number of retrieved records from the selected databases and the process of screening and selecting studies can be viewed in the PRISMA Flow Diagram (Moher et al., 2009, see Figure 1). Next, the first and third author screened the full-text of records that had been assessed as eligible based on their abstracts. The quantified agreement between the raters was high (97% agreement, Cohen’s *k*=0.91; Landis and Koch, 1977). In case of disagreement, the second author assessed the study and discussions were held until agreement was reached. When the final dataset of included studies and their reports was decided upon, the first author independently extracted and coded data available according to the Data Extraction Form (see Supplementary Data Sheet S3). Discussions regarding the data extraction, including the study categorization, were held between the three authors to ensure consistency. Data extracted from each included study were, e.g., author(s), year of publication, method, study setting (country of origin; industry), and key findings.

### Quality Assessment

Quality assessment of the included intervention studies was conducted utilizing the recognized NICE checklist for intervention studies (National Institute for Health and Care Excellence, 2012, based on Spencer et al., 2003; Jackson et al., 2006). The study quality was primarily assessed by the first author followed by discussions among the authors, revealing no discrepancy between the authors’ ratings. A summative quality score was coded for each study as ++, +, or − based on the assessed quality of study population, allocation of participants, outcomes, analyses, and internal and external validity. The highest quality rating (++) indicated low risk of bias, and this rating was given to studies that fulfilled all or most checklist criteria (and it was unlikely that the study conclusions would have been different if the few unfulfilled criteria had been fulfilled). Similarly, a moderate-quality rating (+) indicated moderate risk of bias and this rating was given to studies in which some of the checklist criteria had been fulfilled. The conclusions would likely have remained the same if unfulfilled criteria had been fulfilled, or if poor descriptions of criteria had been adequate. Finally, the lowest quality rating (−) indicated high risk of bias. Studies that received this rating fulfilled few or no criteria and the study conclusions would likely have been different if the missing criteria had been fulfilled.

### Calculation of Effect Sizes and Statistical Analyses

The effect sizes of the interventions were calculated by Review Manager 5.4.1 software (The Cochrane Collaboration, 2020) for the primary outcome under study (i.e., work engagement). Data from all the publications that provided eligible post-test or follow-up data on overall work engagement measured by the UWES-scale (i.e., no sub-scale data considered) were extracted from the study reports by the first author and then double checked and entered into the Review Manager by the third author. Both the weighted mean difference (WMD) and the standardized mean difference (SMD) were calculated as appropriate for the continuously distributed outcome using a random effects model. The random effects model was chosen based on guidelines and recommendations provided by, e.g., APA Publication Manual (Cohen, 1988) for increased interpretability and generalizability. Endpoint continuous data for intervention completers were used in these calculations. With regard to eligible studies with more than two arms, only the intervention-arm and the control-arm that received no intervention were considered in the meta-analysis. If measures of variance of outcomes could not be found in the study publications or through calculations, the corresponding authors of the identified publications were contacted with data requests. If the missing data could not be retrieved, the study was excluded from the meta-analysis. Substantially skewed data (where the standard deviation was greater than double the mean value) were not entered in the meta-analysis. The impact of statistical heterogeneity on the meta-analysis was assessed by quantifying inconsistency among the studies with the *I*^2^ Index test (Deeks et al., 2008). This test describes the percentage of the variability in effect estimates that is due to heterogeneity rather than sampling error (chance). All calculated *I*^2^-values were deemed acceptable, however, all over 50% indicating the proportion of the variation in point estimates due to among-study differences being moderate to large. A sensitivity analysis was conducted to test the robustness of the performed analysis and related findings. Only the interventions that retrieved the highest quality rating (++) in the methodological quality assessment exercise were included in this sensitivity analysis. The extracted data also allowed for three post-hoc sub-group analyses; two of them according to two of the explored potential underlying mechanisms and one of them only including studies that applied the short version of the UWES (Schaufeli et al., 2006). The extracted data also allowed for a meta-analysis of pooled effect sizes for role performance (secondary outcome).

## Results

### Studies Retrieved for the Systematic Review and Meta-Analysis

The total number of records originally identified in the systematic database searches was 1,988. After duplicates were removed, the abstracts of 1,468 unique records were screened according to the eligibility criteria. During this abstract screening process, an additional 1,341 records were excluded, leaving us with 127 records. Main reasons for exclusion of records at this stage were that they were not intervention studies, did not have work engagement as the primary outcome of the study, and/or were not targeted at working individuals. Following a careful assessment of full-text articles, the final number of articles included in the systematic review was 30, of which one contained two included studies (Gordon et al., 2018), resulting in 31 independent studies (see Figure 1 and Table 1). The main reasons for exclusion of articles that were assessed for eligibility in full-text were that they were judged to have a top-down rather than a bottom-up approach, emphasized physiological resources related to lifestyle and bodily health rather than psychosocial resources related to the interaction between the individual and the workplace, and/or did not use the UWES-scale for measuring work engagement. Also, a few of the excluded intervention studies were organizational level studies that did not target workers at the individual level. The number of studies that contributed with data to the meta-analysis was 24, and the sample size at baseline for these studies can be viewed in Table 2.

**Table 1:** Summary of core characteristics and main findings of the included studies in the systematic review and meta-analysis.

Author (year)	Quality appraisal^a^	Study setting	UWES-scale (version)^b^	Quantitative design	Qualitative design^c^	Foci	Approach	Format^d^	Reported finding (WE)^e^	Included in M-A^f^
Akkermans et al. (2015, Sample 2)	+	Netherlands; Industry not mentioned	Overall (short)	Two-armed (non-randomized) intervention	N/A	Career self-management	Tailored	F2F	Increased	Yes
Bakker and van Wingerden (2020)	++	Netherlands; Mixed industries	Overall (short)	Two-armed (non-randomized) intervention	N/A	Strengths use	Universal	F2F	Increased	Yes
Bernburg et al. (2016)	++	Germany; Health care	Overall (short)	Two-armed (randomized) intervention	N/A	Ego resources	Universal	F2F	No effect	Yes
Coo and Salanova (2018)	+	Spain; Health care	Overall + sub-scales; vigor, dedication, absorption (short)	Two-armed (non-randomized) intervention	N/A	Ego resources	Universal	F2F	Increased	Yes
Costantini et al. (2019)	−	Italy; Health care	Overall (short; 5 items)	One-armed intervention	N/A	Strengths use	Universal	F2F	Increased	No
Dubbelt et al. (2019, Study 2)	+	Netherlands; Education	Overall (short; vigor and dedication-items)	Two-armed (non-randomized) intervention	N/A	Job crafting	Tailored	F2F	Increased	Yes
Dyrbye et al. (2016)	++	United States; Health care	Sub-scale; absorption (long)	Two-armed (randomized) intervention	N/A	Strengths use	Tailored	Online	No effect	No
Gollwitzer et al. (2018)	++	Germany; Health care	Overall (short)	Three-armed (randomized) intervention	N/A	Ego resources	Universal	Online	Increased	Yes
Gordon et al. (2018, Study 1)	+	Netherlands; Health care	Overall (short)	Two-armed (non-randomized) intervention	N/A	Job crafting	Tailored	F2F	Increased	Yes
Gordon et al. (2018, Study 2)	+	Netherlands; Health care	Overall (short)	Two-armed (non-randomized) intervention	N/A	Job crafting	Tailored	F2F	Increased	Yes
Kloos et al. (2019)	++	Netherlands; Health care	Overall (short)	Two-armed (randomized) intervention	Open-ended feedback (in questionnaire)	Strengths use	Universal	Online	No effect	Yes
Kuijpers et al. (2020)	+	Netherlands; Health care	Sub-scales; vigor, dedication, absorption (short)	Two-armed (non-randomized) intervention	N/A	Job crafting	Universal	F2F	Increased	Yes^*^
Lases et al. (2016)	+	Netherlands; Health care	Overall (short)	Two-armed (non-randomized) intervention	Open-ended feedback (phone interviews)	Ego resources	Universal	F2F	No effect	Yes
Mastenbroek et al. (2015)	+	Netherlands; Health care	Overall (short)	Two-armed (non-randomized) intervention	Face-to-face interviews	Job crafting	Universal	F2F	No effect	Yes
Meyers & van Woerkom (2017)	++	Netherlands; Mixed industries	Overall (short)	Two-armed (non-randomized) intervention	N/A	Strengths use	Universal	F2F	No effect	No
Muuraiskangas et al. (2016)	−	Finland; Engineering	Overall (short)	One-armed	Phone interviews	Ego resources	Tailored	Online	No effect	No
Oude Hengel et al. (2012)	++	Netherlands; Engineering	Overall + sub-scales; vigor, dedication, absorption (short)	Two-armed (randomized) intervention	N/A	Ego resources	Tailored	F2F	No effect	Yes
Ouweneel et al. (2013)	++	Netherlands; Mixed industries	Overall (short)	Two-armed (non-randomized) intervention	N/A	Strengths use	Tailored	Online	No effect	Yes
Peláez et al. (2020)	+	Spain; Engineering	Overall (short)	Two-armed (non-randomized) intervention	Open-ended question face-to face	Strengths use	Universal	F2F	Increased	Yes
Peláez Zuberbuhler et al. (2020)	+	Spain; Engineering	Overall (short)	Two-armed (non-randomized) intervention	Open-ended question face-to face	Strengths use	Universal	F2F	Increased	Yes
Sakuraya et al. (2016)	−	Japan; Mixed industries	Overall (short)	One-armed	N/A	Job crafting	Universal	F2F	Increased	No
Sakuraya et al. (2020)	++	Japan; Mixed industries	Overall (short)	Two-armed (randomized) intervention	N/A	Job crafting	Universal	F2F	No effect	Yes
Seppälä et al. (2020)	+	Finland; Education	Overall (short)	Two-armed (non-randomized) intervention	Open-ended feedback (in questionnaire)	Job crafting	Tailored	F2F	Decreased	Yes
van Berkel et al. (2014)	++	Netherlands; Education	Overall (long)	Two-armed (randomized) intervention	Face-to-face interviews	Ego resources	Tailored	F2F	No effect	Yes
van Wingerden et al. (2016)	+	Netherlands; Health care	Overall (short)	Two-armed (non-randomized) intervention	N/A	Job crafting	Universal	F2F	Increased	Yes
van Wingerden et al. (2017a)	+	Netherlands; Education	Overall (short)	Two-armed (non-randomized) intervention	N/A	Job crafting	Tailored	F2F	Increased	No
van Wingerden et al. (2017b)	+	Netherlands; Education	Overall (short)	Two-armed (non-randomized) intervention	N/A	Job crafting	Tailored	F2F	No effect	Yes
van Wingerden et al. (2017c)	+	Netherlands; Education	Overall (short)	Four-armed (non-randomized) intervention	Face-to-face interviews	Job crafting	Tailored	F2F	Increased	No
Verweij et al. (2016)	+	Netherlands; Health care	Overall + sub-scales; vigor, dedication, absorption (long)	Two-armed (non-randomized) intervention	Open-ended feedback face-to-face/in evaluation forms	Ego resources	Tailored	F2F	Increased (only dedication)	Yes
Vuori et al. (2012)	++	Finland; Mixed industries	Overall (short)	Two-armed (randomized) intervention	N/A	Career self-management	Tailored	F2F	Increased	Yes
Vuori et al. (2019)	++	Finland; Mixed industries	Overall (short)	Two-armed (randomized) intervention	N/A	Career self-management	Tailored	F2F	Increased	Yes

a Quality appraisal: ++=high-quality score; +=moderate-quality score; and −=low-quality score.

b UWES-scale: Overall=work engagement measured as a high-order construct; Sub-scale(s)=sub-components of work engagement measured (i.e., dedication, absorption, and/or vigor). Version: short=9 items; long=17 items.

c Qualitative design: N/A=not applicable.

d Format: F2F=face-to-face.

e Reported finding: WE=work engagement.

f M-A: M-A=meta-analysis.

* Data on overall work engagement retrieved from another source than the actual published report.

**Table 2:** Sample size at baseline of studies included in the meta-analysis.

Author (year)	Sample size at baseline (intervention)	Sample size at baseline (control)	Sample size at baseline (total)
Akkermans et al. (2015, Sample 2)	72	41^a^	113
Bakker and van Wingerden (2020)	54	48^a^	102
Bernburg et al. (2016)	26	28^a^	54
Coo and Salanova (2018)	19	15^a^	34
Dubbelt et al. (2019, Study 2)	60	59^a^	119
Gollwitzer et al. (2018)	41	47^a^	88
Gordon et al. (2018, Study 1)	48	71^a^	119
Gordon et al. (2018, Study 2)	32	26^a^	58
Kloos et al. (2019)	79	49^a^	128
Kuijpers et al. (2020)	45	54^a^	99
Lases et al. (2016)	22	47^a^	69
Mastenbroek et al. (2015)	21	9^a^	30
Oude Hengel et al. (2012)	171	122^a^	293
Ouweneel et al. (2013)	878	1330^a^	2,208
Peláez et al. (2020)	35	25^a^	60
Peláez Zuberbuhler et al. (2020)	23	15^a^	38
Sakuraya et al. (2020)	138	143^a^	281
Seppälä et al. (2020)	21	19^a^	40
van Berkel et al. (2014)	129	126^a^	255
van Wingerden et al. (2016)	43	24^a^	67
van Wingerden et al. (2017b)	45	30^a^	75
Verweij et al. (2016)	43	20^a^	63
Vuori et al. (2012)	365	341^b^	706
Vuori et al. (2019)	355	337^b^	692

a No intervention control group.

b Other intervention control group.

### Methodological Quality of the Included Studies

The quality assessment exercise was challenging due to scant reporting in several studies. Poor descriptions of population, allocation of participants (if applicable), and statistical analyses performed were especially common shortcomings of the study reports assessed. Based on the reported information, three studies received a low-quality score, indicating high risk of bias. In comparison, 16 studies received a moderate-quality score, indicating moderate risk of bias, and 12 received a high-quality score, indicating low risk of bias. The overall quality score for each study can be viewed in Table 1.

### Characteristics of the Included Studies

#### General Characteristics

In Table 1, core characteristics and main findings of the 31 systematically reviewed studies (total sample size, *n*=6,708) are summarized. Study sample sizes ranged between 34 (Coo and Salanova, 2018) and 2,208 (Ouweneel et al., 2013). Akkermans et al. (2015) have two samples, of which only one (sample 2) was included in the review since sample 1 consisted of students. The gender distribution between samples varied (4–99.32% male), as did average age of participants at baseline (27–58.1years for those studies which provided this data, *n*=29). The included studies were conducted in several different industries, such as the education sector (Dubbelt et al., 2019, Study 2; van Berkel et al., 2014; van Wingerden et al., 2017a,b,c; Seppälä et al., 2020), the engineering sector (Oude Hengel et al., 2012; Muuraiskangas et al., 2016; Peláez et al., 2020; Peláez Zuberbuhler et al., 2020), and the health care sector – the most frequently represented industry (*n*=13). It was also relatively common that the included studies were based on a sample composed of participants from mixed industries (*n*=7). Industry was not reported in one of the included studies (Akkermans et al., 2015). Regarding the geographical context, a clear majority of the included studies were conducted in Europe (*n*=28). Of the European studies, as many as 18 studies were conducted in The Netherlands. Another European country, Finland, was also quite well represented with four studies. Only three studies were conducted outside Europe, in Japan (*n*=2) and United States (*n*=1). Program duration varied extensively across studies, ranging from half a day (Meyers and van Woerkom, 2017) to 10months (Mastenbroek et al., 2015). The intervention program in 10 studies lasted 1month or less, 14 studies more than 1month but less than 3months, and seven studies 3months or longer. Regarding publication year, none of the included studies were published prior to 2012. The majority of the included studies was conducted in the last 5years, peaking in 2016 (*n*=7). Regarding the publication outlet, the most common journals were *Journal of Vocational Behavior* (*n*=4), *Frontiers in Psychology* (*n*=3), *Journal of Happiness Studies* (*n*=3), *European Journal of Work and Organizational Psychology* (*n*=2), *Journal of Occupational Health Psychology* (*n*=2), *and Human Resource Management* (*n*=2). The rest of the represented journals published one article each. The included interventions were categorized in different groups to explore potential mechanisms underlying their effectiveness:

#### Potential Mechanisms Underlying the Intervention Effectiveness

##### Intervention Foci

Intervention focus, i.e., the content of the intervention program and the workplace resources in focus for development, varied. We categorized the interventions according to focus into four different groups based on the proactive bottom-up approaches put forth by Bakker (2017).

The first group of interventions had a strength-based approach (*n*=8) and was underpinned by positive psychology frameworks. These interventions were designed to encourage the participants to identify, develop, and use their inner strengths and talents, with the intention to make them function optimally, perform well, and engage in their work. The majority (Ouweneel et al., 2013; Meyers and van Woerkom, 2017; Kloos et al., 2019; Bakker and van Wingerden, 2020; Peláez et al., 2020; Peláez Zuberbuhler et al., 2020) included development of psychological capital or its sub-components (i.e., self-efficacy, hope, optimism, and resilience) for the promotion of work engagement. Other resources that were developed in this group of interventions included self-esteem, assertiveness, and positive affect.

The second group of interventions was focused on mobilizing ego resources (*n*=8). Participants proactively developed their inherent energetic, affective, or cognitive resources. Six interventions were based on various forms of mindfulness (e.g., mindfulness-based stress reduction, mind fitness training, and mindful vitality in practice), two of them combined with other training components (such as training in strengths use, stress management, and obtaining social support). The two remaining interventions evaluated a stress reduction program (including mental contrasting) and an empowerment program.

Three studies (Vuori et al., 2012, 2019; Akkermans et al., 2015) shared the third focus: career self-management. Participants conducted various exercises, in which they reflected on and developed their own career skills and competencies (e.g., assertiveness), work ability (e.g., social skills and networking), and employability (e.g., find interesting new tasks). A trustful and supportive environment was crucial as the intervention involved active learning, brainstorming, social modeling, and roleplaying. Participants practiced self-goal setting, drew up personal work-related plans, and prepared for potential setbacks.

Job crafting was the fourth and most dominant focus identified in the retrieved studies (*n*=12). The participants were encouraged to make proactive changes in resources external to themselves, i.e., in their job characteristics and social relationships at work. The participants took part of information on and practiced general personal job crafting strategies, after which they developed and implemented their own personal crafting plans. One intervention trained participants in job crafting by means of visual arts. To increase effectiveness, studies added experiential learning techniques (Gordon et al., 2018, Study 1 & Study 2; Dubbelt et al., 2019), exercises aimed at aligning job tasks with inner strengths and abilities (Mastenbroek et al., 2015; van Wingerden et al., 2016, 2017a,b,c; Kuijpers et al., 2020), and cognitive training to redefine one’s work situation (Sakuraya et al., 2016, 2020).

The interventions focusing on strengths use, mobilizing ego resources, and career self-management all share the characteristic that they predominantly developed resources inherent in the individual employees themselves. In contrast, the core of the interventions focused on job crafting was to develop resources that resided in the participants’ social work context and the way work was organized.

##### Intervention Approach

The intervention studies were also categorized in two different groups depending on whether they applied a universal approach, or an approach tailored to the target group’s specific needs.

In the interventions applying a universal approach (*n*=15), the intervention program was generic, and the exercises, methods, and techniques used could equally well have been delivered to other groups of workers. While participants in most of these interventions were encouraged to decide for themselves what resources they wanted to develop during the program, the design and implementation of the program components were not specifically tailored to the work context of the participants and population-specific needs and preferences were not targeted.

The tailored interventions (*n*=16) were at least partially crafted for the targeted population. The whole intervention program was tailored in eight interventions. That is, the intervention design was informed by interviews and meetings with managers and workers from participating organizations, and in some cases also with other stakeholders, pre-assessment questionnaires, and/or a robust literature on population-specific needs and preferences. Three other interventions were tailored in the sense that they included active teaching and learning methods. This meant that the participants’ own knowledge and work context, not lectures, were the starting point for the interventions. The intervention content was thus very specific and applicable to the participants’ real-life work situation. Similarly, a tailored aspect was described in five interventions, such as the inclusion of practical examples in training sessions or text and pictures in booklets that were adapted to the population in question.

##### Intervention Format

Intervention format refers to how the interventions were delivered to the participants. We categorized the interventions according to format in two different groups.

First, five interventions were delivered through an online format (Ouweneel et al., 2013; Dyrbye et al., 2016; Muuraiskangas et al., 2016; Gollwitzer et al., 2018; Kloos et al., 2019). These were app- or web-based and focused on individual exercises that the participants completed online, tasks that they undertook in their everyday working life, and educational elements. One intervention included gamified aspects (e.g., use of avatars and tailored automatic feedback). In another intervention, participants were offered the possibility to share their experiences by engaging in online group discussions with other participants and an e-coach.

The second group of interventions was clearly dominant. Here, interventions were delivered face-to-face (*n*=26). Seven interventions were facilitated by the researchers, four by trainers working in the organization, 11 by external experts, and three by both researchers and external experts. Regardless of facilitator, the intervention core was training sessions conducted in a group setting. Participants were educated in bottom-up strategies that were discussed and applied individually, in pairs, or in larger groups. In 23 interventions, participants were additionally assigned with minor individual tasks and exercises or provided with coaching to increase effectiveness. For example, participants could receive a booklet containing learning materials, exercises, or space to write down individual goals or reflections.

### Evidence Statements

#### Effects on Work Engagement

All studies included in the systematic review (*n*=31) applied quantitative data analysis approaches; 10 of these also applied qualitative data analysis methods. The effect on overall work engagement (measured as a higher-order construct by the UWES-scale; Schaufeli et al., 2006) was reported in 30 studies (see Table 1). Among them, increased work engagement was reported in 16 studies (ca 53% of the studies); however, in the study of Vuori et al. (2012), work engagement only increased in one-side testing. Lack of effect was reported in 13 studies (ca 43% of the studies) and a significant decrease in work engagement was reported in one study (Seppälä et al., 2020). In total, five studies (Oude Hengel et al., 2012; Dyrbye et al., 2016; Verweij et al., 2016; Coo and Salanova, 2018; Kuijpers et al., 2020) reported effects on at least one of the three sub-components of work engagement as measured by the UWES-scale (Schaufeli et al., 2006). The effect on vigor was reported in four studies: vigor increased in one study (Coo and Salanova, 2018) and did not change in three studies (Oude Hengel et al., 2012; Verweij et al., 2016; Kuijpers et al., 2020). Dedication was measured in four studies, of which three reported a positive significant effect (Verweij et al., 2016; Coo and Salanova, 2018; Kuijpers et al., 2020) and one no significant effect (Oude Hengel et al., 2012). Finally, the effect on absorption was reported in five studies, of which two reported a positive effect (Coo and Salanova, 2018; Kuijpers et al., 2020) and three no effect (Oude Hengel et al., 2012; Dyrbye et al., 2016; Verweij et al., 2016).

**Figure 2:**
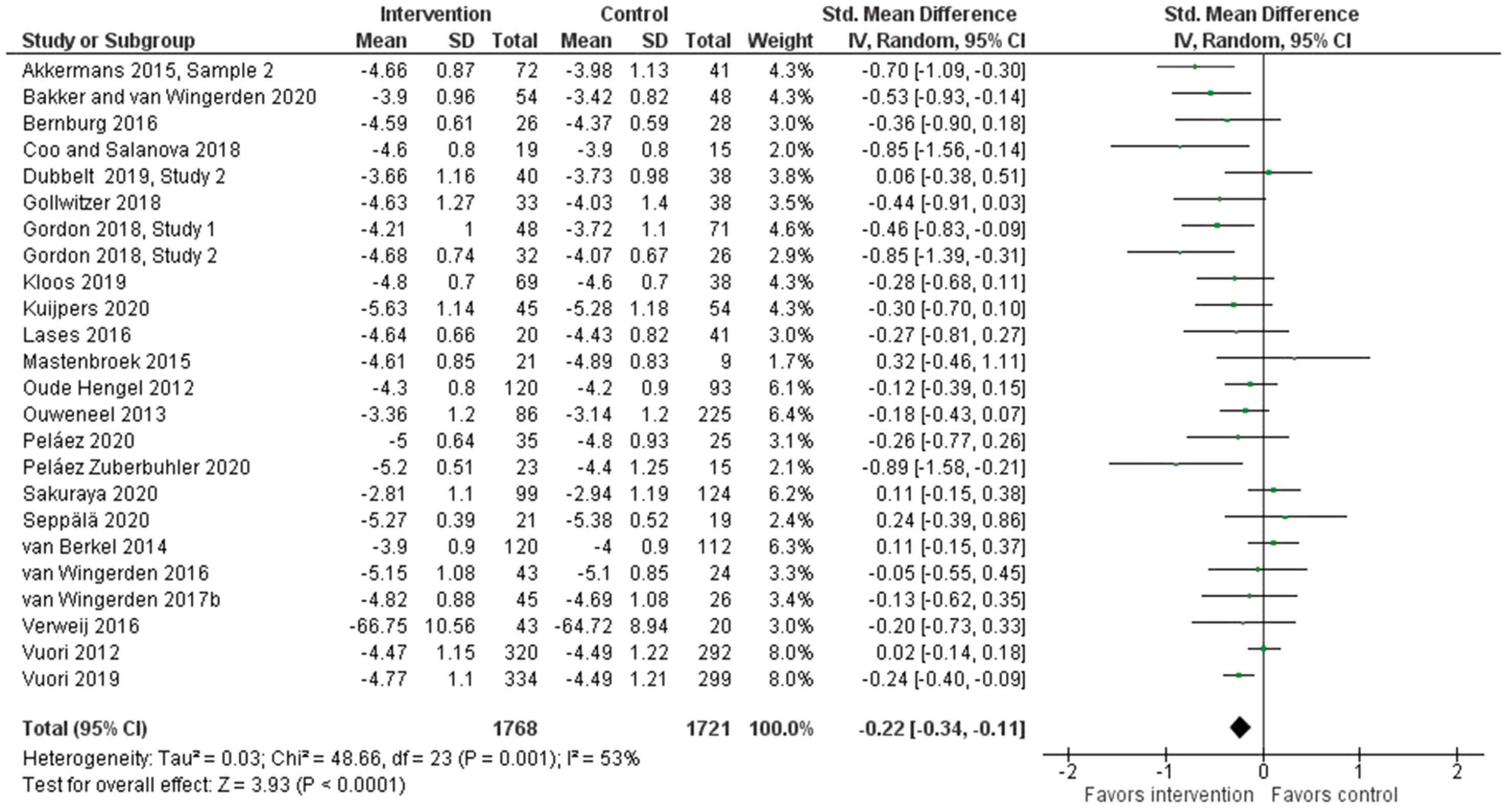
Effect of bottom-up, resource-developing interventions *versus* no-intervention controls on work engagement.

In the meta-analysis with pooled data comparing the effects of interventions to no-intervention (*n*=22) or other intervention (Vuori et al., 2012, 2019) controls, work engagement (as measured by the short or long version of the UWES-scale) showed a small but promising statistically significant improvement (24 interventions, SMD: −0.22, 95% CI: −0.34 to −0.11; Figure 2). The analysis showed moderate heterogeneity (*I*^2^ =53%), indicating some inconsistency of the calculated effect size. In a sub-group analysis only including the interventions using the short version of the UWES-scale, the pooled effect size remained nearly the same (23 interventions, WMD: −0.21, 95% CI: −0.32 to −0.10), with *I*^2^ =55%.

*Evidence statement 1: The synthesized evidence shows that bottom-up interventions aimed at promoting work engagement by developing workplace resources are effective. The evidence base on the effectiveness of interventions for the promotion of overall work engagement is both stronger and more promising than that for the promotion of sub-components of work engagement. The conducted meta-analysis revealed a small but promising statistically significant improvement in overall work engagement across the identified interventions*.

#### Effects on Secondary Outcomes: Satisfaction and Performance

A few of the studies included in the systematic review, all conducted with work engagement as the primary outcome, also reported the effectiveness of the intervention on secondary outcomes. Among these additional outcomes, dimensions of satisfaction and performance were frequently reported.

The intervention effect on dimensions of satisfaction was reported in seven studies, of which all except one reported increased satisfaction. The intervention effect on job satisfaction was reported in three studies, of which two (Bernburg et al., 2016; Kloos et al., 2019) reported a statistically significant increase in job satisfaction. In contrast, Dyrbye et al. (2016) reported a significant decrease in job satisfaction and additionally no statistically significant effect on satisfaction with work-life balance. Finally, a statistically significant positive effect was reported in one study each on work satisfaction (Lases et al., 2016), career satisfaction (Dubbelt et al., 2019, Study 2), basic need satisfaction (van Wingerden et al., 2017a), and life satisfaction (Meyers and van Woerkom, 2017).

The intervention effect on dimensions of performance was reported in nine studies. A statistically significant increase in performance was reported in all of them, in terms of task performance (Dubbelt et al., 2019, Study 2), adaptive, task and contextual (but not objective) performance (Gordon et al., 2018, Study 1 & Study 2), and (in−/extra-) role performance (van Wingerden et al., 2016, 2017a,b,c; Coo and Salanova, 2018; Peláez et al., 2020; Peláez Zuberbuhler et al., 2020). All these measures of performance were assessed with a variety of measurements, such as the Healthy & Resilient Organization (HERO) questionnaire (Salanova et al., 2012), the in-role performance scale (Williams and Anderson, 1991), and Goodman and Svyantec's (1999) task and contextual performance scale.

**Figure 3:**
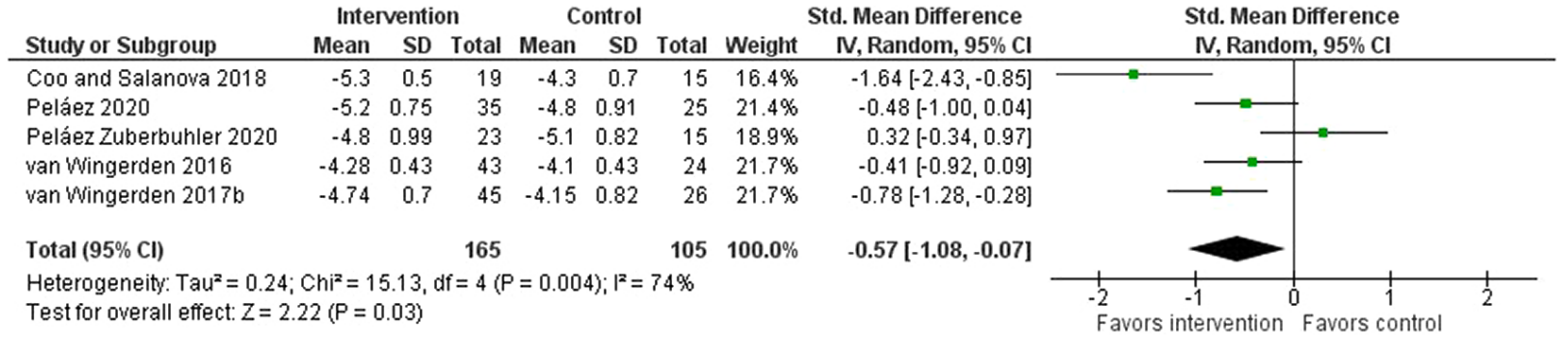
Effect of bottom-up, resource-developing interventions *versus* no-intervention controls on role performance (secondary outcome).

A meta-analysis was conducted on a sub-set of studies that reported the intervention effect on role performance specifically and that provided eligible information to compute pooled effect sizes. In this meta-analysis, role performance showed a moderate to large and statistically significant improvement (five interventions, SMD: −0.57, 95% CI: −1.08 to −0.07; Figure 3). The analysis showed high heterogeneity (*I*^2^ =74%), indicating high inconsistency of the calculated effect size.

*Evidence statement 2: In the synthesis, scattered evidence was found on the effectiveness of bottom-up interventions in promoting satisfaction at work, as well as scarce but promising evidence for promoting performance. The conducted meta-analysis on the intervention effectiveness on role performance showed a moderate to large and statistically significant improvement – but also revealed a high heterogeneity, which makes for caution in interpreting the results. The results on these secondary outcomes were found even though the primary intervention aim was to promote work engagement by developing workplace resources. This indicates that bottom-up interventions for the promotion of work engagement also have potential to yield other positive outcomes in addition to work engagement, and therefore future workplace intervention research should include measurements of, e.g., satisfaction and performance – applying standardized and comparable instruments*.

#### Comparing the Effectiveness of the Interventions Based on Their Foci

**Figure 4:**
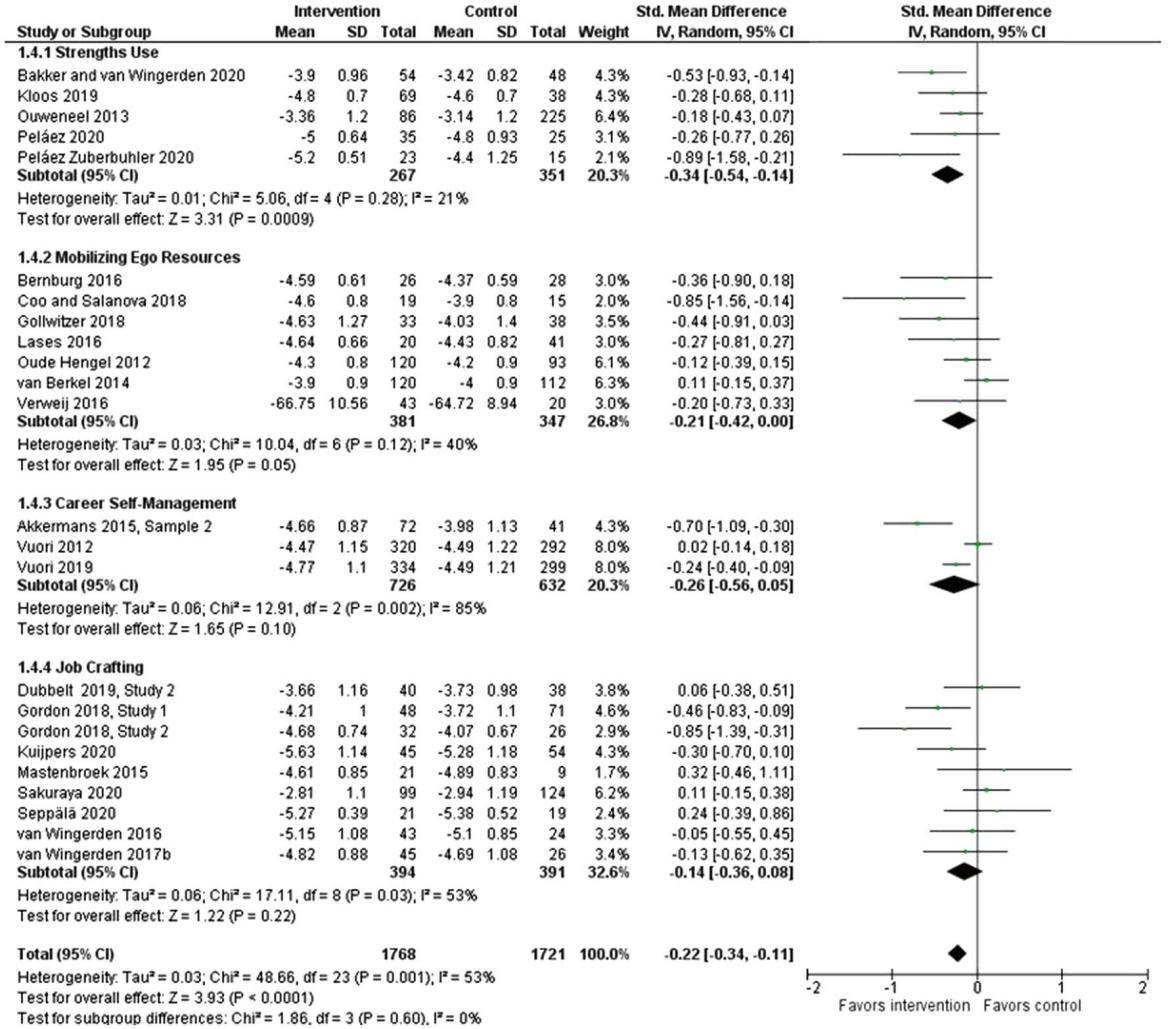
Effect of bottom-up, resource-developing interventions *versus* no-intervention controls on work engagement according to intervention foci.

To investigate the most effective intervention foci in relation to the primary outcome under study, further analysis was carried out as part of the meta-analysis exercise for those controlled interventions that were categorized as focusing on strengths use (Ouweneel et al., 2013; Kloos et al., 2019; Bakker and van Wingerden, 2020; Peláez et al., 2020; Peláez Zuberbuhler et al., 2020); mobilizing ego resources (*n*=7); career self-management (Vuori et al., 2012, 2019; Akkermans et al., 2015); and job crafting (*n*=9). The strengths use category showed a promising and statistically significant effect on work engagement (SMD: −0.34, 95% CI: −0.54 to −0.14). The category mobilizing ego resources had at most a small statistically significant effect (SMD: −0.21, 95% CI: −0.42 to 0.00). In contrast, the two remaining categories did not show any statistically significant effect: career self-management (SMD: −0.26, 95% CI: −0.56 to 0.05) and job crafting (SMD: −0.14, 95% CI: −0.36 to 0.08). See Figure 4.

*Evidence statement 3: The analysis comparing the pooled data on effectiveness between four intervention categories indicates that intervention focus is a mechanism underlying the intervention effect on work engagement, providing convincing evidence for the category of interventions focusing on strengths use. The analysis also supports the intervention category focusing on mobilizing ego resources, while the two categories encompassing interventions with a career self-management or a job crafting focus failed to show any pooled significant effects*.

#### Comparing the Effectiveness of the Interventions Based on Their Approach

**Figure 5:**
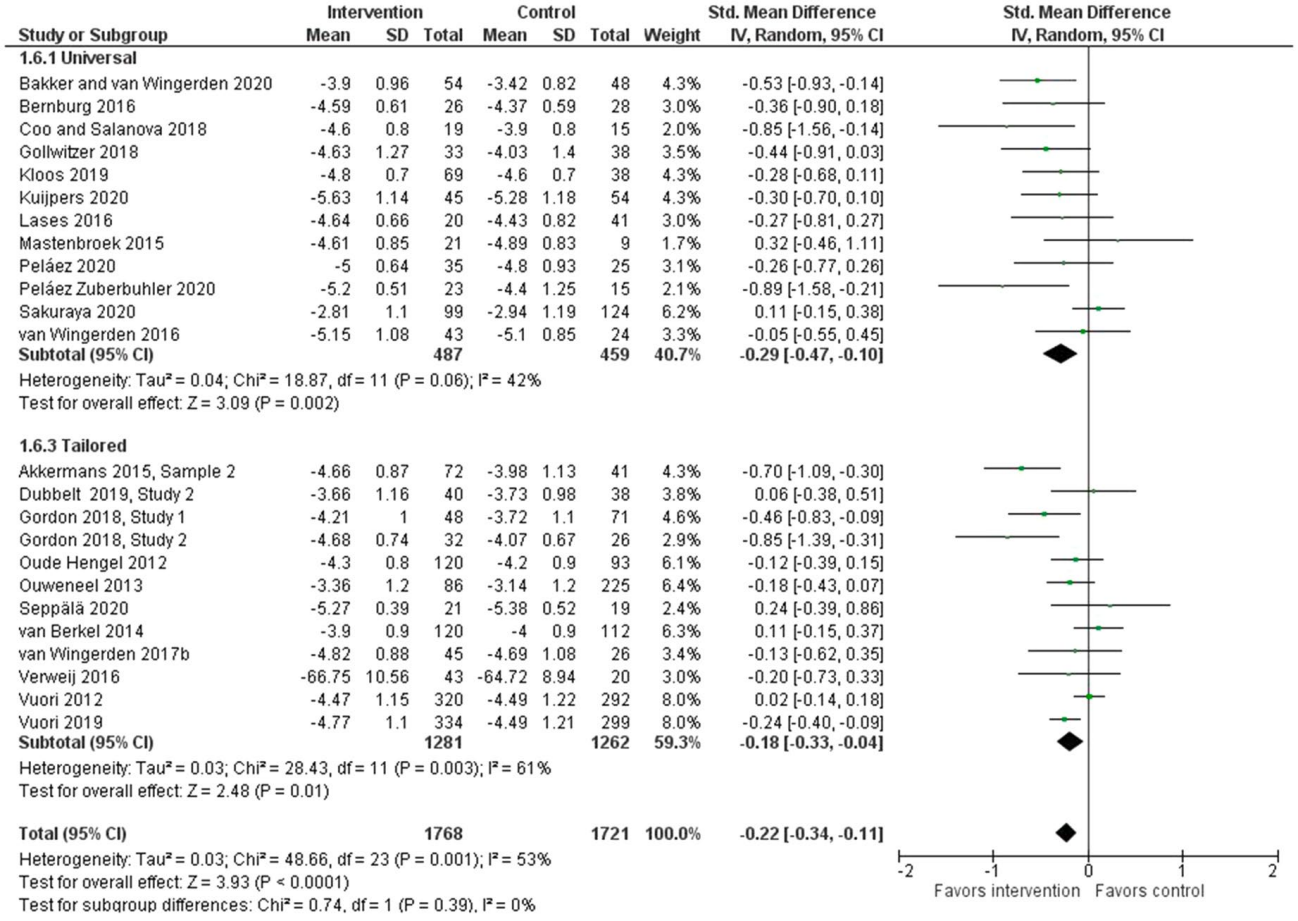
Effect of bottom-up, resource-developing interventions *versus* no-intervention controls on work engagement according to intervention approach.

The work engagement interventions comparing intervention participants with no-intervention participants were also compared according to intervention approach. While interventions with both universal and tailored programs had a statistically significant positive effect on work engagement, the effect of interventions with a universal approach was larger (*n*=12, SMD: −0.29, 95% CI: −0.47 to −0.10) compared to that of interventions with a tailored approach (*n*=12, SMD: −0.18, 95% CI: −0.33 to −0.04). See Figure 5.

*Evidence statement 4: Based on the meta-analysis comparing the evidenced effect sizes between two intervention approaches, it can be argued that the approach of the interventions delivered is a central mechanism underlying the intervention effectiveness on work engagement, with a larger effect size for a universal approach compared to a tailored approach*.

#### Sensitivity Analysis

To investigate the robustness of the analyses performed as part of the meta-analysis and related findings, a sensitivity analysis was performed. Here, only the interventions deemed rigorous in their study design and with low risk of bias (i.e., scored with ++) in the quality assessment exercise were included. Based on the sensitivity analysis, we argue that the findings from the meta-analysis are robust, despite the inclusion of interventions with varying design and quality. Considering the high-quality interventions only, the overall effect of interventions on work engagement remained statistically significant (10 interventions, SMD: −0.14, 95% CI: −0.27 to −0.01), indicating a small but promising positive effect on work engagement among the intervention participants compared to control conditions. The heterogeneity (*I*^2^) of the sensitivity analysis was 52%.

#### Participant Experiences of the Interventions

Ten of the 31 reviewed intervention studies adopted mixed methods, meaning that they combined quantitative measures with qualitative data, which entailed reporting on participants’ experiences of and reflections on the intervention design, outcome, or both. Participant experiences were gathered through interviews and open-ended questions in questionnaires and training sessions.

Specifically, participant experiences related to the intervention design were reported in five studies (van Berkel et al., 2014; Lases et al., 2016; Muuraiskangas et al., 2016; Kloos et al., 2019; Seppälä et al., 2020). The participant experiences were predominantly positive in three of the studies (van Berkel et al., 2014; Lases et al., 2016; Muuraiskangas et al., 2016). The interventions in these studies were described as innovative, interesting, and useful, and the content was found to be easy to understand and appreciated by the participants regardless of used format (i.e., online or face-to-face). Seppälä et al. (2020) mostly reported negative experiences, such as perceived flaws in information, quality and structure of the intervention, and the professional skills of the trainers. In the study conducted by Kloos et al. (2019), participant experiences of the intervention design were mixed, with some experiencing that the content was relevant while others did not, and the majority perceived the intervention set-up as an area of improvement. In the studies conducted by Muuraiskangas et al. (2016) and Seppälä et al. (2020), participants experienced difficulties in balancing participation in the intervention with work-related obligations, as these interventions were conducted during work hours.

Similarly, all mixed-methods studies except for Seppälä et al. (2020) reported on how the participants experienced the intervention outcome. All these studies reported that the majority of participants experienced the effect of the intervention, if any, as positive. For example, participants experienced enhanced work engagement (Mastenbroek et al., 2015), well-being (Muuraiskangas et al., 2016; Verweij et al., 2016; Peláez et al., 2020), energy (van Berkel et al., 2014; Verweij et al., 2016), and performance (Peláez et al., 2020, Peláez Zuberbuhler et al., 2020) post-intervention. Further, the participants described how the intervention had supported them in developing crucial workplace resources at multiple levels, both resources that the intervention specifically targeted, and other ones. Such resources included awareness of own thoughts, emotions, and behavior (Mastenbroek et al., 2015; Muuraiskangas et al., 2016; Verweij et al., 2016; Kloos et al., 2019; Peláez et al., 2020; Peláez Zuberbuhler et al., 2020). Participants also experienced that they developed resources in terms of self-acceptance, self-esteem, and compassion toward oneself and others as additional positive effects of the interventions (Mastenbroek et al., 2015; Verweij et al., 2016). In six studies (van Berkel et al., 2014; Lases et al., 2016; Verweij et al., 2016; van Wingerden et al., 2017c; Peláez et al., 2020; Peláez Zuberbuhler et al., 2020), the qualitative results on the intervention outcomes to a great extent supported the quantitative ones, while the reported qualitative results in three studies clearly differed from the quantitative in that they were more positive (Mastenbroek et al., 2015; Muuraiskangas et al., 2016; Kloos et al., 2019).

*Evidence statement 5: There is promising evidence that bottom-up interventions aimed at promoting work engagement by developing workplace resources are well received among the participants and generate positive experiences among them*.

## Discussion

The present study aimed to systematically review the evidence base of interventions conducted for the promotion of work engagement by developing workplace resources from bottom-up. Further, the aim was to perform a statistical meta-analysis of the eligible evidence, as well as to explore mechanisms underlying the evidenced effectiveness, if any.

The results lend support to the effectiveness of the investigated interventions for the promotion of overall work engagement. This is in accordance with multiple theoretical frameworks, such as the COR theory (Hobfoll, 1989; Halbesleben et al., 2014), the JD-R model (Demerouti et al., 2001), and the broaden-and-build theory (Fredrickson, 2001). Specifically, the systematic review showed that 53% of the 30 studies that measured work engagement as a higher-order construct reported an improvement. This finding was corroborated in the meta-analysis, which was based on 24 studies and demonstrated a small but positive statistically significant effect on overall work engagement. This positive intervention effect is suggested to be widely applicable, at least in European settings, as it was found by systematically reviewing and meta-analyzing studies conducted in various industries and across various groups of workers.

Unfortunately, we only found a small number of studies investigating the intervention effect on sub-components of work engagement (as defined in the UWES-scale; Schaufeli et al., 2006). Therefore, it would not have been feasible to conduct a sub-analysis on the sub-components in the meta-analysis. The systematic review found scattered evidence for the effect on vigor, dedication, and absorption. According to a previous review of the meaning, antecedents, and outcomes of engagement, measuring sub-components of work engagement tends to yield more complex results than measuring overall work engagement (Bailey et al., 2017). Similarly, we found scattered evidence for a positive intervention effect on the secondary outcome satisfaction at work, and scarce but promising evidence for intervention effectiveness on the secondary outcome performance at work. Hence, we encourage future workplace intervention research to include these outcomes and measure them using standardized and comparable instruments.

The meta-analysis of the interventions according to intervention foci, which were based on the individual bottom-up approaches suggested by Bakker (2017), showed that strengths use and mobilizing ego resources interventions both had a positive statistically significant effect on work engagement. In contrast, career self-management and job crafting interventions did not. The failure to find a significant pooled effect for interventions focused on career self-management is likely due to lack of power, which in turn is the result of high heterogeneity (*I*^2^ =85%) and of there being few studies in this group (only three studies had this focus). However, the sub-group difference between intervention foci in this analysis was not statistically significant. As previously noted by Knight et al. (2017), one explanation for this may be heterogeneity within the sub-groups. Although we did our best in the current review to classify the interventions according to their most dominant focus, we acknowledge that they seldom had one focus only. For example, job crafting interventions included self-goal setting, which is an individual self-management approach (Bakker, 2017). Another potential explanation may be that the categorization of bottom-up approaches proposed by Bakker (2017) is not optimal for categorizing bottom-up interventions. However, this study still highlights that interventions focused on strengths use and mobilizing ego resources are more effective in promoting work engagement than interventions focused on career self-management and job crafting.

The meta-analysis of the intervention effectiveness according to approach showed that both universal and tailored interventions had a statistically significant effect on work engagement compared to control conditions. Further, a statistically significant sub-group difference between intervention approach was found in this analysis, where universal intervention programs were more promising than tailored ones. Although it may be less theoretically attractive, two obvious strengths of taking a universal approach are that it increases generalizability and that it is less time-consuming. Interestingly, in studies where a tailored approach was applied, this was usually highlighted as a strength of the study. At the same time, it was rarely explained on what basis a tailored study approach was developed and it may be that a universal approach would have been at least equally effective in at least some of these studies. The studies that apply a tailored intervention approach also varied extensively regarding the degree to which they were tailored. While the whole intervention program was tailored in some studies, only aspects of the intervention program were tailored in others. It might be that considerable effort has to be made to map the targeted populations’ needs and preference (e.g., conducting a pilot study) and that the intervention needs to be substantially tailored for its effectiveness to increase.

Finally, as part of the systematic review, we examined qualitative data from 10 mixed-methods studies to summarize participant experiences of the intervention design and outcomes. We found that the participants in most of these studies generally appreciated the intervention design. For example, the participants reported that the program content was easy to understand and experienced as useful and interesting. It should be noted though that in all interventions, participants were responsible for initiating and making changes in their own workplace resources. Simply experiencing that ones’ own proactivity is supported and valued can on its own be motivating and thus induce positive feelings toward the design of the intervention. Additionally, in some mixed-methods studies, the participants described the experienced outcomes in more positive terms in the qualitative data than in the quantitative. We can only speculate why this was the case, but it is possible that the participants felt obligated to provide more positive answers in the qualitative data since these data were often gathered through interviews or meetings occurring face-to-face, while the quantitative data were based on anonymous responses. Further, participants reported that they also experienced positive effects other than those intended in the program, such as developing additional resources. Hence, when participants learn, practice, and implement individual bottom-up approaches in work engagement interventions, it seems that the effects even go beyond the desired outcomes.

### Reported Limitations Among the Included Studies

The included studies reported several limitations. Commonly reported shortcomings of the interventions were reliance on self-reports (risk of common method variance), small sample size or high dropout rates (risk of low statistical power), limited generalizability of the study findings (focus on certain industrial and geographical contexts and groups of workers) and that the results were short-term ones (no information on long-term effects). Intervention studies with no comparator and studies with non-randomized intervention and comparator groups often reported these study characteristics as important limitations.

### Limitations of the Present Study

The systematic review and meta-analysis have several limitations. First, the data were collected from studies with varying design and characteristics, also revealing moderate to high inconsistency based on high heterogeneity. Further, our study highlighted risks of reporting bias. Some of the studies included in the systematic review lacked the required information to be included in the meta-analysis (e.g., two arms, means and standard deviation values, and measurement points) but many more provided insufficient descriptions of the study design, sample, and procedure – all of which complicated the assessment of study quality and publication bias. Further, several of the studies included in the meta-analysis were based on a low sample size and thus reduced the statistical power. The lack of statistical significance in some of the findings is probably the result of a combination of small effect sizes and lack of statistical power due to the low number of studies, many of which included small samples. All these factors limited the extent to which conclusions can be drawn from this study regarding the evidenced effectiveness of interventions. However, in order to nuance the information on the evidence identified, as well as to test the robustness of the findings from the meta-analysis exercise, we performed several sub-group analyses. For example, the sensitivity analysis that included only high-quality studies showed a lower but still statistically significant pooled effect on overall work engagement. The reason behind a lowered pooled effect size estimate among the high-quality studies only compared to all included studies could be explained by an on average smaller difference between the intervention and control group in relation to the measured outcome, which in turn implies a slightly weakened relevant effect in practice among these studies. Not only does this call for more intervention studies applying high-quality research design and methods, but it also points out the need for a more nuanced examination of the mechanisms underlying the effectiveness of the studies aiming to promote work engagement.

The second limitation pertains to the categorizations of the included studies. While we did our best to classify the studies in a meaningful way that would further the understanding of how work engagement can be promoted, there is always a risk of mis-categorization due to inconsistency in how information is reported.

A third limitation is that we only included studies that measured work engagement using the UWES-scale (Schaufeli et al., 2006). Although this scale is widely used in the work engagement literature (Bailey et al., 2017; Shuck et al., 2017; Kelders et al., 2020), a recurring criticism concerns its robustness, which is argued to be weakened due to the three-factor structure (Wefald et al., 2012). At the same time, applying use of the UWES-scale as one of the eligibility criteria for this study could be viewed as a strength. One reason for this is that the validity and reliability of the UWES-scale are supported in several studies and in several settings (Schaufeli, 2014). It is also likely that an inclusion of the studies that we excluded on this basis would have aggravated the work with this systematic review and meta-analysis to the extent that the meaningfulness and robustness of the study results had been diminished.

### Implications for Research and Practice

We provide researchers with a checklist that could be used when conducting future studies on bottom-up work engagement interventions (see Table A1). Future intervention research and practice can build upon the aggregated results of our systematic review and meta-analysis in at least three different ways. First, robustness of study findings should be ensured in future bottom-up intervention studies investigating the effect on work engagement. Here, ensuring robustness especially entails ensuring that the study sample is representative of the investigated population, the statistical power is sufficient, and a comparison group is included. Further, the participants should be allocated randomly, or baseline differences between the intervention and the comparison group should at least be controlled for. In the current study, only 12 of the included studies were rated with the highest quality score. For example, a statistically significant increase in work engagement was reported in a clear majority of the systematically reviewed job crafting interventions, while the aggregated results in our meta-analysis showed that this dominant category of intervention focus had no statistically significant effect on work engagement. Moreover, one third of the intervention studies that focused on the promotion of work engagement through job crafting was conducted by van Wingerden et al. (2016, 2017a,b,c) and the resemblance between these studies is high. It is our interpretation that these ground-breaking studies set the tone for most of the subsequent studies that shared this intervention focus, which illustrates the danger in relying on the results of single intervention studies, especially if they can be associated with methodological flaws and risk of bias. From a practical point of view, this learning is also relevant for practitioners, since it suggests that popular practice does not necessarily constitute best practice.

Second, more studies investigating the effects of bottom-up interventions on sub-components of work engagement are warranted. Such studies could deepen our understanding of how bottom-up interventions aimed at promoting sub-components of work engagement stand in comparison with those aimed at promoting overall work engagement. However, based on the synthesized evidence, practitioners are guided to educate, facilitate, and encourage individual bottom-up approaches that promote the overall work engagement of employees.

Third, the evidence retrieved from the meta-analysis suggests that future intervention research should apply universal approaches rather than tailored ones. In practice, these results can be interpreted to imply that similar training, methods, and techniques should be applied to all kinds of employees when organizations want to facilitate the process in which employees learn, practice, and eventually use bottom-up approaches for the development of workplace resources.

## Conclusion

In conclusion, our results evidenced a small but promising intervention effect on overall work engagement. Furthermore, this systematic review and meta-analysis sheds light on the underlying mechanisms of bottom-up, resource-developing interventions that successfully promote work engagement. Based on our findings, we advocate the use of a universal approach and a focus on strengths use or mobilizing ego resources to increase intervention effectiveness. Scholars within the wide and interdisciplinary field of work engagement interventions can benefit from our checklist covering recommendations for future research endeavors to ensure increased evidence robustness and knowledge advances made.

## Data Availability Statement

The original contributions presented in the study are included in the article/Supplementary Material, and further inquiries can be directed to the corresponding author.

## Author Contributions

JMB formulated the aim of the study and the applied eligibility criteria, conducted database searches, screening and selection, coding, and quality assessment, synthesized the study data, prepared the published work, wrote the initial draft, revised and edited the manuscript, as well as acquired the financial support for the publication of this work. PB participated in the discussions around selection and coding of the retrieved data, in addition to contributing to the preparation of the published work, specifically with critical reviews and revisions of the various versions of the manuscript. AKF participated in the final selection of the included studies and related coding exercises, applied statistical techniques to analyze the study data, and contributed to the preparations of the published work, specifically with critical reviews and revisions of the various versions of the manuscript. All authors contributed to the article and approved the submitted version.

## Funding

This research work was funded by the Finnish Work Environment Fund (grant number: 200264) and Svensk-Österbottniska Samfundet (a regional research and development funding instrument).

## Conflict of Interest

The authors declare that the research was conducted in the absence of any commercial or financial relationships that could be construed as a potential conflict of interest.

## Publisher’s Note

All claims expressed in this article are solely those of the authors and do not necessarily represent those of their affiliated organizations, or those of the publisher, the editors and the reviewers. Any product that may be evaluated in this article, or claim that may be made by its manufacturer, is not guaranteed or endorsed by the publisher.

## Appendix

**TABLE A1:** Checklist for future bottom-up intervention studies on work engagement.

Optimize the internal validity:
Aim for a feasible sample size
Include a control group – preferably apply a randomized controlled design
Report and control for baseline differences between the intervention and the control group
Report how well the sample characteristics matched the population characteristics
Report the dropout rate
Calculate the statistical power and report effect sizes
Report long-term effects of the intervention
If possible, include qualitative measures to answer the question of why and how the intervention worked/did not work and to explore potential unintended effects
Optimize the external validity:
Recruit participants from several organizations and occupational groups to increase generalizability
Use standardized and comparable instruments for primary and secondary outcomes
Contribute to under-researched topics:
Investigate intervention effects on the sub-components of work engagement
Study relevant sub-groups
Conduct interventions focused on self-management
Deliver interventions online
Ask the participants about their experiences of both the intervention design and outcomes
Conduct interventions in other contexts than health care
Conduct interventions based on samples from other continents than Europe
