# Supplementary material for: Bottom-Up Interventions Effective in Promoting Work Engagement: A Systematic Review and Meta-Analysis
Source: Front Psychol. 2021 Sep 8;12:730421. doi: 10.3389/fpsyg.2021.730421 (PMC8456101; doi:10.3389/fpsyg.2021.730421)
Supplement: Supplementary file 2 [file Data_Sheet_2.DOCX]

**Supplementary Data Sheet S2 – Search Strategies**

| **Platform/Database** | Search (S) |
| --- | --- |
| **Web of Science** | **S1**  TS=(“work engagement” OR “employee engagement”)  *Timespan=2000-2020*  *Search language=English*  *Document Type=Article* |
|  | **S2**  AB=(“work engagement” OR “employee engagement”)  *Timespan=2000-2020*  *Search language=English*  *Document Type=Article* |
|  | **S3**  TI=(“work engagement” OR “employee engagement”)  *Timespan=2000-2020*  *Search language=English*  *Document Type=Article* |
|  | **S4**  TS=(resource* OR strength* OR “job craft*” OR “proactiv*” OR “positive psychology” OR “person-cent*” OR “person cent*” OR participat* OR (bottom-up AND (job OR work OR design OR re-design OR redesign)))  *Timespan=2000-2020*  *Search language=English*  *Document Type=Article* |
|  | **S5**  AB=(resource* OR strength* OR “job craft*” OR “proactiv*” OR “positive psychology” OR “person-cent*” OR “person cent*” OR participat* OR (bottom-up AND (job OR work OR design OR re-design OR redesign)))  *Timespan=2000-2020*  *Search language=English*  *Document Type=Article* |
|  | **S6**  TI=(resource* OR strength* OR “job craft*” OR “proactiv*” OR “positive psychology” OR “person-cent*” OR “person cent*” OR participat* OR (bottom-up AND (job OR work OR design OR re-design OR redesign)))  *Timespan=2000-2020*  *Search language=English*  *Document Type=Article* |
|  | **S7**  TS=(intervention* OR program* OR initiative* OR action* OR “randomi?ed controlled trial*” OR RCT OR “clinical trial*”)  *Timespan=2000-2020*  *Search language=English*  *Document Type=Article* |
|  | **S8**  AB=(intervention* OR program* OR initiative* OR action* OR “randomi?ed controlled trial*” OR RCT OR “clinical trial*”)  *Timespan=2000-2020*  *Search language=English*  *Document Type=Article* |
|  | **S9**  TI=(intervention* OR program* OR initiative* OR action* OR “randomi?ed controlled trial*” OR RCT OR “clinical trial*”)  *Timespan=2000-2020*  *Search language=English*  *Document Type=Article* |
|  | **S10**  S#1 OR S#2 OR S#3 |
|  | **S11**  S#4 OR S#5 OR S#6 |
|  | **S12**  S#7 OR S#8 OR S#9 |
|  | **S13**  S#10 AND S#11 AND S#12 |
| **EBSCO/ Academic Search Complete** | **S1**  SU=(“work engagement” OR “employee engagement”)  *Search modes - Boolean/Phrase*  *Scholarly (Peer Reviewed) Journals*  *Publication Date=2000-2020*  *Document type=Article*  *Search language=English* |
|  | **S2**  AB=(“work engagement” OR “employee engagement”)  *Search modes - Boolean/Phrase*  *Scholarly (Peer Reviewed) Journals*  *Publication Date=2000-2020*  *Document type=Article*  *Search language=English* |
|  | **S3**  TI=(“work engagement” OR “employee engagement”)  *Search modes - Boolean/Phrase*  *Scholarly (Peer Reviewed) Journals*  *Publication Date=2000-2020*  *Document type=Article*  *Search language=English* |
|  | **S4**  SU=(resource* OR strength* OR “job craft*” OR “proactiv*” OR “positive psychology” OR “person-cent*” OR “person cent*” OR participat* OR (bottom-up AND (job OR work OR design OR re-design OR redesign)))  *Search modes - Boolean/Phrase*  *Scholarly (Peer Reviewed) Journals*  *Publication Date=2000-2020*  *Document type=Article*  *Search language=English* |
|  | **S5**  AB=(resource* OR strength* OR “job craft*” OR “proactiv*” OR “positive psychology” OR “person-cent*” OR “person cent*” OR participat* OR (bottom-up AND (job OR work OR design OR re-design OR redesign)))  *Search modes - Boolean/Phrase*  *Scholarly (Peer Reviewed) Journals*  *Publication Date=2000-2020*  *Document type=Article*  *Search language=English* |
|  | **S6**  TI=(resource* OR strength* OR “job craft*” OR “proactiv*” OR “positive psychology” OR “person-cent*” OR “person cent*” OR participat* OR (bottom-up AND (job OR work OR design OR re-design OR redesign)))  *Search modes - Boolean/Phrase*  *Scholarly (Peer Reviewed) Journals*  *Publication Date=2000-2020*  *Document type=Article*  *Search language=English* |
|  | **S7**  SU=(intervention* OR program* OR initiative* OR action* OR “randomi?ed controlled trial*” OR RCT OR “clinical trial*”)  *Search modes - Boolean/Phrase*  *Scholarly (Peer Reviewed) Journals*  *Publication Date=2000-2020*  *Document type=Article*  *Search language=English* |
|  | **S8**  AB=(intervention* OR program* OR initiative* OR action* OR “randomi?ed controlled trial*” OR RCT OR “clinical trial*”)  *Search modes - Boolean/Phrase*  *Scholarly (Peer Reviewed) Journals*  *Publication Date=2000-2020*  *Document type=Article* |
|  | **S9**  TI=(intervention* OR program* OR initiative* OR action* OR “randomi?ed controlled trial*” OR RCT OR “clinical trial*”)  *Search modes - Boolean/Phrase*  *Scholarly (Peer Reviewed) Journals*  *Publication Date=2000-2020*  *Document type=Article* |
|  | **S10**  S#1 OR S#2 OR S#3 |
|  | **S11**  S#4 OR S#5 OR S#6 |
|  | **S12**  S#7 OR S#8 OR S#9 |
|  | **S13**  S#10 AND S#11 AND S#12 |
| **EBSCO/Business Source Ultimate** | **S1**  SU=(“work engagement” OR “employee engagement”)  *Search modes - Boolean/Phrase*  *Scholarly (Peer Reviewed) Journals*  *Publication Date=2000-2020*  *Document type=Article*  *Search language=English* |
|  | **S2**  AB=(“work engagement” OR “employee engagement”)  *Search modes - Boolean/Phrase*  *Scholarly (Peer Reviewed) Journals*  *Publication Date=2000-2020*  *Document type=Article*  *Search language=English* |
|  | **S3**  TI=(“work engagement” OR “employee engagement”)  *Search modes - Boolean/Phrase*  *Scholarly (Peer Reviewed) Journals*  *Publication Date=2000-2020*  *Document type=Article*  *Search language=English* |
|  | **S4**  SU=(resource* OR strength* OR “job craft*” OR “proactiv*” OR “positive psychology” OR “person-cent*” OR “person cent*” OR participat* OR (bottom-up AND (job OR work OR design OR re-design OR redesign)))  *Search modes - Boolean/Phrase*  *Scholarly (Peer Reviewed) Journals*  *Publication Date=2000-2020*  *Document type=Article*  *Search language=English* |
|  | **S5**  AB=(resource* OR strength* OR “job craft*” OR “proactiv*” OR “positive psychology” OR “person-cent*” OR “person cent*” OR participat* OR (bottom-up AND (job OR work OR design OR re-design OR redesign)))  *Search modes - Boolean/Phrase*  *Scholarly (Peer Reviewed) Journals*  *Publication Date=2000-2020*  *Document type=Article*  *Search language=English* |
|  | **S6**  TI=(resource* OR strength* OR “job craft*” OR “proactiv*” OR “positive psychology” OR “person-cent*” OR “person cent*” OR participat* OR (bottom-up AND (job OR work OR design OR re-design OR redesign)))  *Search modes - Boolean/Phrase*  *Scholarly (Peer Reviewed) Journals*  *Publication Date=2000-2020*  *Document type=Article*  *Search language=English* |
|  | **S7**  SU=(intervention* OR program* OR initiative* OR action* OR “randomi?ed controlled trial*” OR RCT OR “clinical trial*”)  *Search modes - Boolean/Phrase*  *Scholarly (Peer Reviewed) Journals*  *Publication Date=2000-2020*  *Document type=Article*  *Search language=English* |
|  | **S8**  AB=(intervention* OR program* OR initiative* OR action* OR “randomi?ed controlled trial*” OR RCT OR “clinical trial*”)  *Search modes - Boolean/Phrase*  *Scholarly (Peer Reviewed) Journals*  *Publication Date=2000-2020*  *Document type=Article* |
|  | **S9**  TI=(intervention* OR program* OR initiative* OR action* OR “randomi?ed controlled trial*” OR RCT OR “clinical trial*”)  *Search modes - Boolean/Phrase*  *Scholarly (Peer Reviewed) Journals*  *Publication Date=2000-2020*  *Document type=Article* |
|  | **S10**  S#1 OR S#2 OR S#3 |
|  | **S11**  S#4 OR S#5 OR S#6 |
|  | **S12**  S#7 OR S#8 OR S#9 |
|  | **S13**  S#10 AND S#11 AND S#12 |
| **EBSCO/APA PsycInfo** | **S1**  SU=(“work engagement” OR “employee engagement”)  *Search modes - Boolean/Phrase*  *Peer Reviewed*  *Published Date and Publication year=2000-2020*  *Publication Type=Peer Reviewed Journal*  *Language=English* |
|  | **S2**  AB=(“work engagement” OR “employee engagement”)  *Search modes - Boolean/Phrase*  *Peer Reviewed*  *Published Date and Publication year=2000-2020*  *Publication Type=Peer Reviewed Journal*  *Language=English* |
|  | **S3**  TI=(“work engagement” OR “employee engagement”)  *Search modes - Boolean/Phrase*  *Peer Reviewed*  *Published Date and Publication year=2000-2020*  *Publication Type=Peer Reviewed Journal*  *Language=English* |
|  | **S4**  SU=(resource* OR strength* OR “job craft*” OR “proactiv*” OR “positive psychology” OR “person-cent*” OR “person cent*” OR participat* OR (bottom-up AND (job OR work OR design OR re-design OR redesign)))  *Search modes - Boolean/Phrase*  *Peer Reviewed*  *Published Date and Publication year=2000-2020*  *Publication Type=Peer Reviewed Journal*  *Language=English* |
|  | **S5**  AB=(resource* OR strength* OR “job craft*” OR “proactiv*” OR “positive psychology” OR “person-cent*” OR “person cent*” OR participat* OR (bottom-up AND (job OR work OR design OR re-design OR redesign)))  *Search modes - Boolean/Phrase*  *Peer Reviewed*  *Published Date and Publication year=2000-2020*  *Publication Type=Peer Reviewed Journal*  *Language=English* |
|  | **S6**  TI=(resource* OR strength* OR “job craft*” OR “proactiv*” OR “positive psychology” OR “person-cent*” OR “person cent*” OR participat* OR (bottom-up AND (job OR work OR design OR re-design OR redesign)))  *Search modes - Boolean/Phrase*  *Peer Reviewed*  *Published Date and Publication year=2000-2020*  *Publication Type=Peer Reviewed Journal*  *Language=English* |
|  | **S7**  SU=(intervention* OR program* OR initiative* OR action* OR “randomi?ed controlled trial*” OR RCT OR “clinical trial*”)  *Search modes - Boolean/Phrase*  *Peer Reviewed*  *Published Date and Publication year=2000-2020*  *Publication Type=Peer Reviewed Journal*  *Language=English* |
|  | **S8**  AB=(intervention* OR program* OR initiative* OR action* OR “randomi?ed controlled trial*” OR RCT OR “clinical trial*”)  *Search modes - Boolean/Phrase*  *Peer Reviewed*  *Published Date and Publication year=2000-2020*  *Publication Type=Peer Reviewed Journal*  *Language=English* |
|  | **S9**  TI=(intervention* OR program* OR initiative* OR action* OR “randomi?ed controlled trial*” OR RCT OR “clinical trial*”)  *Search modes - Boolean/Phrase*  *Peer Reviewed*  *Published Date and Publication year=2000-2020*  *Publication Type=Peer Reviewed Journal*  *Language=English* |
|  | **S10**  S#1 OR S#2 OR S#3 |
|  | **S11**  S#4 OR S#5 OR S#6 |
|  | **S12**  S#7 OR S#8 OR S#9 |
|  | **S13**  S#10 AND S#11 AND S#12 |
| **EBSCO/APA PsycArticles** | **S1**  SU=(“work engagement” OR “employee engagement”)  *Search modes - Boolean/Phrase*  *Scholarly (Peer Reviewed) Journals*  *Published Date and Year of Publication=2000-2020*  *Document Type= Journal Article*  *Language=Not Applicable (N/A)* |
|  | **S2**  AB=(“work engagement” OR “employee engagement”)  *Search modes - Boolean/Phrase*  *Scholarly (Peer Reviewed) Journals*  *Published Date and Year of Publication=2000-2020*  *Document Type= Journal Article*  *Language=Not Applicable (N/A)* |
|  | **S3**  TI=(“work engagement” OR “employee engagement”)  *Search modes - Boolean/Phrase*  *Scholarly (Peer Reviewed) Journals*  *Published Date and Year of Publication=2000-2020*  *Document Type= Journal Article*  *Language=Not Applicable (N/A)* |
|  | **S4**  SU=(resource* OR strength* OR “job craft*” OR “proactiv*” OR “positive psychology” OR “person-cent*” OR “person cent*” OR participat* OR (bottom-up AND (job OR work OR design OR re-design OR redesign)))  *Search modes - Boolean/Phrase*  *Scholarly (Peer Reviewed) Journals*  *Published Date and Year of Publication=2000-2020*  *Document Type= Journal Article*  *Language=Not Applicable (N/A)* |
|  | **S5**  AB=(resource* OR strength* OR “job craft*” OR “proactiv*” OR “positive psychology” OR “person-cent*” OR “person cent*” OR participat* OR (bottom-up AND (job OR work OR design OR re-design OR redesign)))  *Search modes - Boolean/Phrase*  *Scholarly (Peer Reviewed) Journals*  *Published Date and Year of Publication=2000-2020*  *Document Type= Journal Article*  *Language=Not Applicable (N/A)* |
|  | **S6**  TI=(resource* OR strength* OR “job craft*” OR “proactiv*” OR “positive psychology” OR “person-cent*” OR “person cent*” OR participat* OR (bottom-up AND (job OR work OR design OR re-design OR redesign)))  *Search modes - Boolean/Phrase*  *Scholarly (Peer Reviewed) Journals*  *Published Date and Year of Publication=2000-2020*  *Document Type= Journal Article*  *Language=Not Applicable (N/A)* |
|  | **S7**  SU=(intervention* OR program* OR initiative* OR action* OR “randomi?ed controlled trial*” OR RCT OR “clinical trial*”)  *Search modes - Boolean/Phrase*  *Scholarly (Peer Reviewed) Journals*  *Published Date and Year of Publication=2000-2020*  *Document Type= Journal Article*  *Language=Not Applicable (N/A)* |
|  | **S8**  AB=(intervention* OR program* OR initiative* OR action* OR “randomi?ed controlled trial*” OR RCT OR “clinical trial*”)  *Search modes - Boolean/Phrase*  *Scholarly (Peer Reviewed) Journals*  *Published Date and Year of Publication=2000-2020*  *Document Type= Journal Article*  *Language=Not Applicable (N/A)* |
|  | **S9**  TI=(intervention* OR program* OR initiative* OR action* OR “randomi?ed controlled trial*” OR RCT OR “clinical trial*”)  *Search modes - Boolean/Phrase*  *Scholarly (Peer Reviewed) Journals*  *Published Date and Year of Publication=2000-2020*  *Document Type= Journal Article*  *Language=Not Applicable (N/A)* |
|  | **S10**  S#1 OR S#2 OR S#3 |
|  | **S11**  S#4 OR S#5 OR S#6 |
|  | **S12**  S#7 OR S#8 OR S#9 |
|  | **S13**  S#10 AND S#11 AND S#12 |
| **Scopus** | **S1**  *SUBJAREA(MEDI OR NURS OR VETE OR DENT OR HEAL OR MULT) SUBJAREA(ARTS OR BUSI OR DECI OR ECON OR PSYC OR SOCI) TITLE-ABS-KEY(“work engagement” OR “employee engagement”) LANGUAGE(english) DOCTYPE(ar) SRCTYPE(j) PUBYEAR AFT 1999* |
|  | **S2**  *SUBJAREA(MEDI OR NURS OR VETE OR DENT OR HEAL OR MULT) SUBJAREA(ARTS OR BUSI OR DECI OR ECON OR PSYC OR SOCI) TITLE-ABS-KEY*(resource* OR strength* OR “job craft*” OR “proactiv*” OR “positive psychology” OR “person-cent*” OR “person cent*” OR participat* OR (bottom-up AND (job OR work OR design OR re-design OR redesign))) *LANGUAGE(english) DOCTYPE(ar) SRCTYPE(j) PUBYEAR AFT 1999* |
|  | **S3**  *SUBJAREA(MEDI OR NURS OR VETE OR DENT OR HEAL OR MULT) SUBJAREA(ARTS OR BUSI OR DECI OR ECON OR PSYC OR SOCI) TITLE-ABS-KEY*(intervention* OR program* OR initiative* OR action* OR “randomi?ed controlled trial*” OR RCT OR “clinical trial*”) *LANGUAGE(english) DOCTYPE(ar) SRCTYPE(j) PUBYEAR AFT 1999* |
|  | **S4**  S#1 AND S#2 AND S#3 |
| **Google Scholar** | **S1**  work engagement = exact phrase  resource strength "job crafting" proactivity "positive psychology" "person centred" "person centered" participatory "bottom up" "bottom-up" intervention program initiative action = at least one word  *All searches performed for the time period 2000-2020*  *Terms searched in the title* |
|  | **S2**  employee engagement = exact phrase  resource strength "job crafting" proactivity "positive psychology" "person centred" "person centered" participatory "bottom up" "bottom-up" intervention program initiative action = at least one word  *Publication year: 2000–2020*  *Terms searched in the title* |
