# Supplementary material for: Bottom-Up Interventions Effective in Promoting Work Engagement: A Systematic Review and Meta-Analysis
Source: Front Psychol. 2021 Sep 8;12:730421. doi: 10.3389/fpsyg.2021.730421 (PMC8456101; doi:10.3389/fpsyg.2021.730421)
Supplement: Supplementary file 3 [file Data_Sheet_3.docx]

**Supplementary Data Sheet S3 – Data Extraction Form**

| **BACKGROUND**  **Reference:**  1. Author(s) (year)  **Version of the UWES-scale:**  1. Short version  2. Long version  **Method:**  1. Quantitative  2. Qualitative  3. Mixed-methods  *Quantitative:*  1. Experimental (two or more arms)  a. randomized  b. non-randomized  2. Non-experimental (one arm)  *Qualitative:*  1. Interviews  2. Open-ended questions  3. Other (mention which) |  | **INTERVENTION**  **Duration between measurement points:**  1. T1–T2 (weeks)  2. T1–T2 (weeks); T3  **Comparator (mention sample size at baseline and at final measurement point for intervention and comparison group):**  1. Control group (no intervention) (*n*/*n*)  2. Comparison group (other intervention) (*n*/*n*)  3. No comparison group (*n*)  **Study Setting:**  1. Country; industry  2. Country; mixed industries  3. Country; industry not mentioned  **Intervention Foci:**  1. Strengths use  2. Mobilizing ego resources  3. Career self-management  4. Job crafting  **Intervention Approach:**  1. Tailored  2. Universal  **Intervention Format:**  1. Face-to-face  2. Online |  | **RESULTS**  **Key Findings (work engagement):**  1. Statistically significant increase  2. Statistically significant decrease  3. No statistically significant effect  **Quality Assessment and Risk of Bias:**  1. Low quality score (–) and high risk of bias  2. Moderate quality score (+) and moderate risk of bias  3. High quality score (++) and low risk of bias |
| --- | --- | --- | --- | --- |
